# *In vitro *anti-plasmodial activity of *Dicoma anomala *subsp. *gerrardii *(Asteraceae): identification of its main active constituent, structure-activity relationship studies and gene expression profiling

**DOI:** 10.1186/1475-2875-10-295

**Published:** 2011-10-11

**Authors:** John VW Becker, Marina M van der Merwe, Anna C van Brummelen, Pamisha Pillay, Bridget G Crampton, Edwin M Mmutlane, Chris Parkinson, Fanie R van Heerden, Neil R Crouch, Peter J Smith, Dalu T Mancama, Vinesh J Maharaj

**Affiliations:** 1 Biosciences, CSIR, PO Box 395, Pretoria, 0001, South Africa; 2 School of Chemistry (Pietermaritzburg), University of KwaZulu-Natal, Private Bag X01, Scottsville, 3209, South Africa; 3 Forestry and Agriculture Biotechnology Institute, University of Pretoria, Pretoria, 0001, South Africa; 4 Ethnobotany Unit, South African National Biodiversity Institute, PO Box 52099, Berea Road, 4007, South Africa/School of Chemistry, University of KwaZulu-Natal, Private Bag X01, Scottsville, 3209, South Africa; 5 Pharmacology Division, Department of Medicine, University of Cape Town, K-45 OMB GSH, Observatory, 7925, South Africa; 6 African Centre for Gene Technologies, PO Box 75011, Lynnwood Ridge, Pretoria, 0040, South Africa

## Abstract

**Background:**

Anti-malarial drug resistance threatens to undermine efforts to eliminate this deadly disease. The resulting omnipresent requirement for drugs with novel modes of action prompted a national consortium initiative to discover new anti-plasmodial agents from South African medicinal plants. One of the plants selected for investigation was *Dicoma anomala *subsp. *gerrardii*, based on its ethnomedicinal profile.

**Methods:**

Standard phytochemical analysis techniques, including solvent-solvent extraction, thin-layer- and column chromatography, were used to isolate the main active constituent of *Dicoma anomala *subsp. *gerrardii*. The crystallized pure compound was identified using nuclear magnetic resonance spectroscopy, mass spectrometry and X-ray crystallography. The compound was tested *in vitro *on *Plasmodium falciparum *cultures using the parasite lactate dehydrogenase (pLDH) assay and was found to have anti-malarial activity. To determine the functional groups responsible for the activity, a small collection of synthetic analogues was generated - the aim being to vary features proposed as likely to be related to the anti-malarial activity and to quantify the effect of the modifications *in vitro *using the pLDH assay. The effects of the pure compound on the *P. falciparum *transcriptome were subsequently investigated by treating ring-stage parasites (alongside untreated controls), followed by oligonucleotide microarray- and data analysis.

**Results:**

The main active constituent was identified as dehydrobrachylaenolide, a eudesmanolide-type sesquiterpene lactone. The compound demonstrated an *in vitro *IC_50 _of 1.865 μM against a chloroquine-sensitive strain (D10) of *P. falciparum*. Synthetic analogues of the compound confirmed an absolute requirement that the α-methylene lactone be present in the eudesmanolide before significant anti-malarial activity was observed. This feature is absent in the artemisinins and suggests a different mode of action. Microarray data analysis identified 572 unique genes that were differentially expressed as a result of the treatment and gene ontology analysis identified various biological processes and molecular functions that were significantly affected. Comparison of the dehydrobrachylaenolide treatment transcriptional dataset with a published artesunate (also a sesquiterpene lactone) dataset revealed little overlap. These results strengthen the notion that the isolated compound and the artemisinins have differentiated modes of action.

**Conclusions:**

The novel mode of action of dehydrobrachylaenolide, detected during these studies, will play an ongoing role in advancing anti-plasmodial drug discovery efforts.

## Background

The most critical problem facing malaria treatment today is resistance to anti-plasmodial drugs. The emerging resistance of *Plasmodium falciparum *to the only affordable anti-malarials, chloroquine and sulphadoxine/pyrimethamine, and now even the last effective class of antimalarials, the artemisinins [1,2], undermines efforts to eliminate or even manage effectively this deadly disease. Hence, there is continued interest in the discovery of bioactive natural compounds with unique structures that may lead to anti-malarial drugs with novel modes of action [3-5].

South Africa boasts a rich biodiversity with more than 22,600 indigenous plants, representing about 9% of all higher plants on Earth [6]. There is also a long history of the use of plants in African traditional healthcare systems. This natural resource and indigenous knowledge, together with the historical success of the plant-derived anti-malarials (*i.e*. quinine, artemisinin) and the universal need for new drugs, prompted a national consortium initiative to discover novel anti-plasmodial agents from South African plants [7].

One of the plants selected for this study was *Dicoma anomala*, an erect, suberect or prostrate herb bearing aromatic semi-woody tubers at the base of a woody subterranean stem. This grassland species is widely distributed in sub-Saharan Africa, and is morphologically diverse, resulting in the recognition of several infraspecific taxa. Two subspecies occur in South Africa: *D. anomala *subsp. *anomala *and *D. anomala *subsp. *gerrardii *[4].

The numerous ethnomedicinal uses of *D. anomala*, which range from the treatment of coughs and colds, fevers, ulcers, dermatosis, venereal diseases, labour pains, dysentery, intestinal parasites, stomach pains, toothache and internal worms, can be linked to several pharmacological properties: anti-bacterial, anti-helmintic, anti-viral, anti-plasmodial, anti-spasmodic, wound healing, analgesic and anti-inflammatory [8-11]. A previous study reported the anti-bacterial and anti-inflammatory properties of the root extracts of *D. anomala *[9]. Another study demonstrated *in vitro *anti-cancer activity of extracts of a related species, *Dicoma capensis *[12,13].

**Figure 1:**
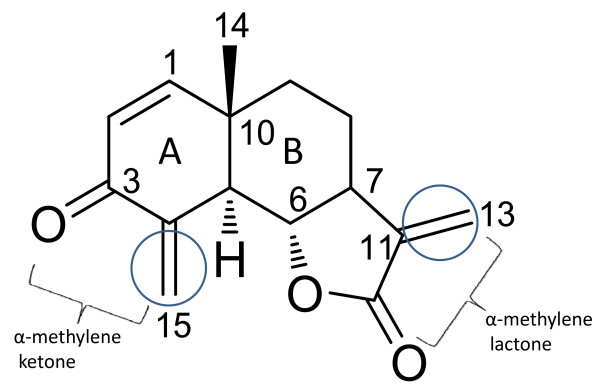
**Chemical structure of the sesquiterpene lactone dehydrobrachylaenolide**. The potential conjugate acceptor sites (circled in blue) and terminology used in the text of this work are indicated.

**Figure 2:**
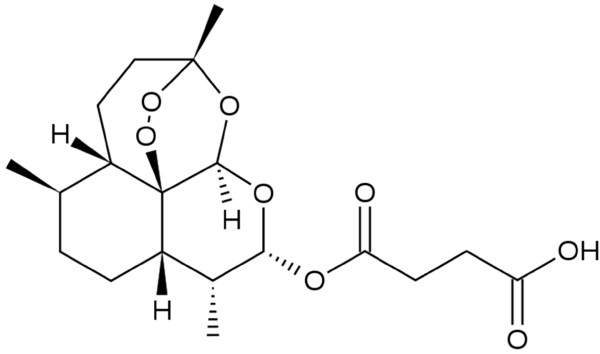
**Chemical structure of the sesquiterpene lactone artesunate**.

Phytochemical investigations of *D. anomala *have identified several classes of secondary metabolites; including acetylenic compounds, phenolic acids, flavonoids, sesquiterpene lactones, triterpenes and phytosterols [14-16]. The investigation of a dichloromethane extract of the roots of *D. anomala *resulted in the isolation of asymmetrical sesquiterpene dimers with potent anti-plasmodial properties [17,18]. Based on this finding and the reported use of *D. anomala *in the treatment of fevers, a primary symptom of malaria [8,9], an ethyl acetate (EtOAc) extract of the roots of *D. anomala *subsp. *gerrardii *was assayed against a chloroquine-sensitive strain of *P. falciparum*. Its activity warranted further investigation and a sesquiterpene lactone, dehydrobrachylaenolide (Figure 1), was isolated by column chromatography and found to show *in vitro *activity [11]. Structure-activity relationship experiments (through the generation and assessment of simple synthetic analogues) confirmed the requirement for activity as the α-methylene lactone moiety, a feature absent in artemisinin anti-malarials (artesunate, Figure 2). The compound was further investigated through transcriptome analysis and comparisons were drawn between the isolated compound and a related sesquiterpene lactone, artesunate (Figure 2). Comparative analysis revealed little overlap and confirmed likely different modes of action.

## Methods

### Plant collection and extraction

Collection of rootstocks of *D. anomala *subsp. *gerrardii *was undertaken in the Brits region of the North West Province of South Africa during February 2008. A voucher (BP 01455) was deposited at the National Herbarium of South Africa (Pretoria), and the identity of this taxon confirmed by Dr E. Retief.

A total of 5.6 kg of fresh rootstock was oven dried (30°C) and ground with a hammer mill to yield 3.7 kg of finely processed plant material. Of this, 3.4 kg of the processed material was consecutively extracted with 3 volumes of EtOAc (15 L) at room temperature over 3 days. The filtered extract was concentrated under reduced pressure on a rotary evaporator at 60°C and further dried in a vacuum desiccator for 96 h to remove all traces of solvent. The extraction yielded 48 g of brown gum (1.3% w/w of dry plant material).

### Isolation and identification of dehydrobrachylaenolide

Solvent partitioning of the crude extract was used to divide the extract into three fractions with different polarities. The extract (1 g) was dissolved in 100 mL of aqueous methanol (MeOH/H_2_O 9:1, v/v) and extracted with hexane (3 × 100 mL). MeOH was removed from the aqueous methanolic layer under vacuum on a rotary evaporator; the remaining solution was diluted to 100 mL with H_2_O and extracted with dichloromethane (DCM) (3 × 100 mL). The DCM layers were combined and dried over anhydrous Na_2_SO_4_, filtered and concentrated under vacuum on a rotary evaporator to give a DCM-soluble fraction. This process was scaled up for the remaining crude extract.

Large-scale purification of the DCM-soluble fraction was performed by liquid chromatography in batches of 5 - 10 g of extract on 400 - 500 g of silica gel 60 (0.063 - 0.2 mm) under reduced pressure, using EtOAc/hexane/DCM/MeOH and acetone/DCM/MeOH gradients. Pooled fractions were combined on the basis of similarity of chemical profile as determined by thin-layer chromatography (TLC). Crude fractions 1A-1L were concentrated under vacuum on the rotary evaporator. Fractions 1D-1H were combined (3.5 g) on the basis of similarity of their TLC profile and subjected to further column chromatography using a 90 cm column packed with silica gel and eluted with an EtOAc/hexane/DCM/MeOH isocratic solvent mixture (9:45:45:1, v/v) to give fractions 2A-2N. The fractions were combined on the basis of similarity of the chemical composition determined by TLC analysis. Further fractionation of 2F (0.4 g) was achieved by means of flash chromatography, using a 40 cm flash column, packed with flash silica gel (silica gel 60) and eluted with a hexane/DCM/EtOAc/MeOH isocratic solvent mixture (45:45:9:1, v/v). This yielded compound 1, which was recrystallized from 2-propanol to yield 400 mg (0.013% w/w of dry plant material) of white orthorhombic crystals.

Compound 1 was identified using standard one- and two-dimensional nuclear magnetic resonance spectroscopic (NMR) experiments; electrospray-ionization mass spectrometry (ESI-MS); X-ray crystallography and by direct comparison with published spectral data [19-21].

NMR analysis was carried out on a Varian 400 MHz Unity spectrometer. The mass spectral data were obtained from a hyphenated HPLC-UV/MS instrument (HPLC Waters Alliance 2690 system, equipped with a Waters 996 photodiode array detector and a triple quadrupole Quattro LC Micro mass spectrometer). The mass spectrometer was set to operate in both the ESI^- ^and ESI^+ ^modes (49 - 478 amu) with a gain of 10; the cone voltage was varied between 20 and 70 eV; the desolvation temperature was 450°C; and the source block temperature was set at 120°C. Crystallography experiments were conducted on an Oxford Diffraction Excalibur-2 CCD area detector diffractometer, using Mo-Kα X-radiation and a graphite-crystal monochromator. Optical rotations were measured in chloroform at room temperature on a Perkin-Elmer 241 polarimeter at 589 nm (Na D-line) using a 1 dm cell.

### Parasite cultures

Asexual *P. falciparum *chloroquine-sensitive 3D7 and D10 cultures, as well as chloroquine-resistant K1 strain cultures, were maintained and synchronized according to established methods [22,23] in an atmosphere consisting of 5% oxygen, 5% carbon dioxide and 90% nitrogen. The parasites were maintained at a 5% haematocrit in RPMI 1640 medium supplemented with 25 mM HEPES, 22 mM glucose, 0.0088% hypoxanthine, 40 ug/mL gentamycin (all Sigma) and 0.5% Albumax (Invitrogen).

### *In vitro *anti-malarial assay

The anti-plasmodial activities of the extracts and pure compounds were determined against the chloroquine-sensitive D10 and the chloroquine-resistant K1 strains via the parasite lactate dehydrogenase (pLDH) assay [24]. All test substances were stored at -20°C prior to testing. Stock solutions of test substances were prepared in 10% MeOH or 10% DMSO (in water), depending on the solubility, and diluted in Millipore water to prepare 2 mg/mL solutions, which were stored at -20°C. Chloroquine diphosphate (Sigma) was used as a reference drug in all experiments. The test substances were evaluated as ten serial two-fold dilutions (0.2 - 100 μg/mL final concentration) in medium (two replicates per concentration point) in duplicate wells. The highest concentration of organic solvent that the parasites were exposed to was 0.5%, which was shown to have no measurable effect on parasite viability [7]. Test solutions were mixed with suspensions of infected human red blood cells to achieve a final haematocrit of 1% and parasitaemia of 2% and incubated for 48 h in microtitre plates (250 uL final volume). Incubation conditions were as described above for the routine maintenance of the *P. falciparum *cultures. The IC_50 _values, serving as a measure for anti-plasmodial activities, were calculated by non-linear regression dose-response analysis using GraphPad Prism v.4.0 software.

### *In vitro *cytotoxicity assay

Compounds were tested for *in vitro *cytotoxicity against a Chinese hamster ovary (CHO) cell line using the MTT [3-(4,5-dimethylthiazol-2-yl)-2,5-diphenyltetrazolium bromide] assay [25]. The CHO cells were cultured in DMEM: Hams F-12 medium (1:1) supplemented with 10% heat-inactivated foetal calf serum (FCS) and gentamycin (0.04 μg/mL) (Highveld Biological, RSA). Samples were dissolved in 10% MeOH. Stock solutions (2 mg/mL) were prepared and were stored at -20°C until use. The samples were added to CHO cells as five ten-fold serial dilutions ranging from 100 - 0.01 μg/mL. A serial dilution of emetine (100 - 0.001 μg/mL) was used as a positive control. Cells were seeded in 96-well plates at a concentration of 10^5^/mL (100 μL per well) and incubated at 37°C for 24 h in a humidified 5% CO_2_-air atmosphere. The medium was subsequently aspirated from the wells and replaced with 200 μL of the test compound dilutions. The microplates were incubated at 37°C for 48 h, after which 25 μL of sterile MTT (5 mg/mL in PBS) was added to each well and incubation was continued for 4 h at 37°C. Formazan crystals formed by viable cells were dissolved in DMSO and quantitated by measuring absorbance at 540 nm in a microtitre plate reader (Cambridge Technologies). Cell viabilities were calculated relative to reference wells containing no test compounds (positive cell controls) and no cells (background controls), plotted against log[test sample] and IC_50 _values derived by non-linear regression analysis using GraphPad Prism v.4.0 software.

### Generation of synthetic analogues of compound 1

Information on the synthesis of compound 1 analogues for structure-activity relationship studies is provided [see Additional file 1].

### Transcriptome analysis

#### Parasite drug treatment and RNA extraction

*P. falciparum *3D7 cultures were treated with 12.5 μM of compound 1, corresponding to an inhibitory concentration of IC_99 _as determined from dose-response curves. A morphological comparison between compound 1 treated versus untreated parasites was performed to assess the effect of treatment and to establish sampling times, by examining the stage-specific progression of parasites across the intra-erythrocytic development cycle. The solvent (DMSO) or compound 1 was added in the window shortly after red blood cell (RBC) invasion and parasite development was monitored 6 hourly over a 48 h period using conventional Giemsa-stained smears. Fresh cultures were scaled up to 50 ml (5% hematocrit, 10% parasitemia), synchronized twice and treated with compound 1 in the window around invasion. Fifteen milliliters of culture was harvested at each of the stages used for transcriptional analyses (2, 6 and 12 hpt). Similar volumes were harvested from untreated *P. falciparum *3D7 control cultures at the same time points. Parallel drug treatments were conducted, serving as biological replicates. RNA was extracted from the frozen erythrocyte pellets as described [26].

#### Universal Reference RNA (URR) pool, cDNA synthesis and hybridization

The URR pool was constructed from mixed-stage cultures as described [26]. cDNA synthesis and hybridizations was as described in [26], with the exception that 40 pmoles of each dye was hybridized to each array, utilising Cy3 (sample) and Cy5 (reference) dyes from GE Healthcare (#RPN5661), specifically optimized for nucleic acid labelling.

#### Microarray data analysis and real-time quantitative PCR (RT-qPCR)

Generation of GenePix results (gpr) files, LIMMA and MARRAY analysis in the R computing environment, calculation of Pearson correlation coefficients and determination of differentially expressed genes were performed as detailed in [26]. RT-qPCR was performed on an Applied Biosystems 7500 Fast cycler (Carlsbad, California, USA) as described [26]. qPCR primers were designed using Primer3 Plus [27] and sequences are available on request. PCR efficiencies for each sample were calculated using LinRegPCR, software described in [28]. Fluorescence data per cycle of each sample is exported from the Applied Biosystems 7500 Fast cycler, from which the efficiencies of individual samples were calculated. These efficiencies were used to calculate relative expression in a mathematical model described by Pfaffl [29]. Gene ontology analyses were performed using the Micro Array Data Interface for Biological Annotation (MADIBA) software [30].

## Results

### Isolation and identification of compound 1

In the search for novel anti-malarials from South African plants, an EtOAc extract of *D. anomala *subsp. *gerrardii *was screened against the chloroquine-sensitive D10 strain of *P. falciparum *using the pLDH assay. The extract exhibited an IC_50 _of 1.4 μg/mL [11].

A phytochemical investigation of this extract resulted in the isolation of a major compound, compound 1. NMR analysis revealed the presence of an *α*-methylene-*γ*-lactone moiety and two fused cyclohexane rings forming the skeleton of the eudesmanolide-type sesquiterpene lactone. The A ring was found to possess a 1,2-endocyclic double bond, a carbonyl at C-3 and an additional exocyclic methylene at C-4. Compound 1 was subsequently identified as 3-oxoeudesma-1,4(15),11(13)-triene-12,6*α*-lide, which was identical to the structure of dehydrobrachylaenolide (Figure 1, see Additional file 2), previously isolated from *Brachylaena transvaalensis *[20], *Hieracium intybaceum *[31] and various *Dicoma *species [16], all of the Asteraceae family. A successful chemical synthesis, which confirmed the structure and absolute configuration of dehydrobrachylaenolide, has been reported [21].

The relative configuration of the asymmetric centres at C-5, C-6 and C-7 was deduced to be *trans *based on the size of the observed ^1^H NMR coupling constants and NOE correlations. This was in good agreement with previously reported configuration data for dehydrobrachylaenolide [16,32], and was subsequently confirmed by X ray crystallography and optical rotation studies [33].

### *In vitro *anti-plasmodial activity of dehydrobrachylaenolide and its synthetic analogues

The eudesmane-type natural compounds are expected to have various biological activities due to their chemical structure [21] but the anti-malarial activity of dehydrobrachylaenolide has not yet been reported. Sesquiterpene lactones are known to have anti-plasmodial activities [34-37] and, therefore, the isolated dehydrobrachylaenolide was tested for potential activity against *P. falciparum*. Toxicity against mammalian CHO cells was used to derive a therapeutic index for the test compound [11]. The results are summarized in Table 1.

**Table 1:** *In vitro *activity of dehydrobrachylaenolide against *P. falciparum*.

Compound	D10 IC_50 _(μM) n = 8	K1 IC_50 _(μM) n = 4	CHO IC_50 _(μM) n = 2	D10 TI	K1 TI
Dehydrobrachylaenolide	1.865 ± 0.254	4.095 ± 0.579	17.199	9.2	4.2

Chloroquine	0.038	0.200	35.800	942	179

D10 = *P. falciparum *- chloroquine-sensitive strain

K1 = *P. falciparum *- chloroquine-resistant strain

CHO = Chinese hamster ovary cells

n = number of technical replicates averaged - each independent experiment performed in duplicate

TI = Therapeutic index i.e. CHO IC_50_/anti-plasmodial IC_50_

The compound had an IC_50 _of 1.865 μM (0.455 μg/mL) against the chloroquine-sensitive D10 strain and 4.095 μM (1.0 μg/mL) against the chloroquine-resistant K1 strain when tested *in vitro *[see Additional file 3]. Dehydrobrachylaenolide can therefore be considered a hit, because it complies with the basic criteria for anti-parasitic drug discovery [38] with an *in vitro *IC_50 _against whole protozoa of ≤ 1 μg/ml and a selectivity of close to ten-fold more against the chloroquine-sensitive parasites than against the CHO cells (see therapeutic index, Table 1).

The working assumption in assessing active functionality was that the levels of unsaturation in the eudesmanolide, in particular the α-methylene lactone within dehydrobrachylaenolide (Figure 1), would be responsible for the observed activity. Several analogues (2 - 6) were synthesized to investigate the structure-activity relationships within dehydrobrachylaenolide and are depicted in Table 2. Due to the time frames involved in obtaining permits for plant collection, plant collection, and metabolite isolation, a semi-synthetic analogue (2) of dehydrobrachylaenolide (1) was generated by reducing the α-methylene appended to the ketone in the A-ring and increasing the unsaturation of this ring. The synthetic analogue (2) generated lacked the anti-malarial potency of the natural product (1), being six-fold less active against the D10 strain. However, this activity was considered sufficient to examine functional group relationships to activity. Reduction of the α-methylene appended to the lactone yielded santonin (3), which was devoid of any significant anti-malarial activity or mammalian toxicity (as measured by activity against CHO) at the upper concentrations of the biological assays. As expected, complete removal of unsaturation in the A-ring resulted in the generation of a compound (4) lacking both activity and toxicity. Re-introduction of the exocyclic methylene to the lactone resulted in a singly unsaturated derivative (5) which displayed moderate anti-malarial activity (about half the potency of the parent (2)). The CHO-based toxicity of this species was, however, substantially reduced (about twenty-fold from the synthetic parent (2) and almost ten-fold from the natural product). The re-introduction of limited unsaturation in the A-ring (6), (tested as a mixture) resulted in no significant improvement in anti-malarial activity with respect to (5), but increased mammalian cell toxicity.

**Table 2:** Analogues generated for determination of active sub-structures.

Structure	D10 strain IC_50_ (μM) n = 2	K1 strain IC_50_ (μM) n = 2	CHO IC_50 _(μM) n = 2
	1.865*	4.095^#^	17.2

	11.5	9.05	7.01

	> 40	NT	> 400

	> 40	NT	> 400

	22.1	NT	124

	16.9	NT	41.9

n = number of technical replicates averaged - each independent experiment performed in duplicate with several independent experiments performed for the parent compound dehydrobrachylaenolide

* n = 8

^# ^n = 4

### Transcriptome analysis of dehydrobrachylaenolide inhibition

#### Culturing, drug treatment and RNA extraction

**Figure 3:**
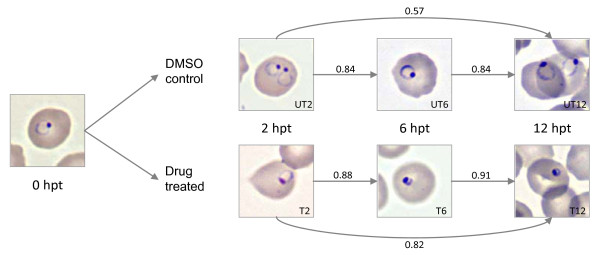
**Morphological analysis of *P. falciparum *3D7 following addition of dehydrobrachylaenolide at three time points and Pearson correlation values between treated and untreated samples**.

A morphological analysis was performed with dehydrobrachylaenolide to ascertain the point during intra-erythrocytic development at which *P. falciparum *3D7 growth was affected. Drug treatments were performed in the window following RBC invasion (i.e. during the early ring stage growth phase). No significant morphological differences were observed between treated and control cultures immediately following drug administration (T = 0), while at 6 h post-treatment (hpt, T = 6) the majority of the treated parasite cultures were already observed to be developing slower than the untreated cultures (Figure 3). Clear differences in morphological development were visible at 12 hpt (T = 12), where virtually all treated parasites were smaller in size and abnormal in shape compared to the control cultures. Treated parasites remained arrested in the ring phase throughout the rest of the 48-hour intraerythrocytic cycle, by the end of which they were observed to be disintegrated, shrivelled or compact. Based on these morphological observations and the apparent rapid effect of dehydrobrachylaenolide on *P. falciparum *development, sampling times for transcriptional analysis were subsequently determined as 2, 6 and 12 hpt. RBC Giemsa-stained smears taken at these time points confirmed the onset of the inhibitory effect of dehydrobrachylaenolide within this timeframe.

#### Gene expression profiling of *P. falciparum *treated with dehydrobrachylaenolide

A microarray reference design experimental strategy, similar to that described in [26] was employed to ascertain global gene expression in parasites perturbed with dehydrobrachylaenolide. Labelled cDNA was hybridized to Operon oligonucleotide microarrays, interrogating 4585 unique genes. All data has been deposited in the NCBI's Gene Expression Omnibus (GEO) database, GEO number GSE29874. Pearson correlation coefficient values for both technical and biological variation were calculated in Microsoft Excel. Technical variation across all arrays was calculated to be 0.89 ± 0.13; whereas biological variation across all slides was calculated as 0.78 ± 0.16. Datasets were collated into untreated (UT_2_, UT_6 _and UT_12_) and treated (T_2_, T_6 _and T_12_) samples at each time point before calculation of Pearson correlation coefficients between drug-treated and control samples. Correlation coefficients suggested that parasites remained in similar states across the different time points after dehydrobrachylaenolide treatment, as can be gauged from coefficients between T_2 _and T_6 _(0.88) and T_6 _and T_12 _(0.91). The correlation between T_2 _and T_12 _was calculated as 0.82, indicating a growth delay close to or around 6 hpt. This notion is strengthened by the fact that Pearson correlation between UT_6 _and T_12 _was 0.70, while the parallel comparison (UT_12 _and T_12_) was calculated at 0.57. For differential gene expression analyses, T_2 _parasites were contrasted to UT_2_, and T_12 _and T_6 _parasites contrasted to UT_6_. A full table of correlation values between all collated samples is provided [see Additional file 4]. A total of 572 unique genes were differentially regulated (cumulatively across all time points) at least two-fold and with a false discovery rate (FDR) cut off of 5% following parasite perturbation with dehydrobrachylaenolide (all genes listed in Additional file 5). The number of differentially regulated genes represents 12.5% of the genes present on the array. Of these, 297 were upregulated (52% of the total), and 275 downregulated (48% of the total). The number of differentially expressed transcripts increased with time following drug addition, as expected from Pearson correlations, which indicates a drug effect on parasite transcription at approximately 6 hpt. Affected transcripts for T_2_, T_6 _and T_12 _were 134, 291 and 378, respectively. Several transcripts were differentially expressed at more than one time point (e.g. T_2 _and T_6 _or T_6 _and T_12_). Due to the cytocidal nature of sesquiterpene lactones [39,40], the high treatment dose (IC_99_) and the rapid time of action of dehydrobrachylaenolide, the transcriptional changes observed at the last time point (T = 12) were regarded as less informative concerning mode of action and were not analysed further. In a related transcriptome investigation of *P. falciparum *parasites treated with lethal doses of artesunate (a derivative of the sesquiterpene lactone artemisinin), time points were harvested after 90 minutes and 3 hours resulting in 398 differentially affected genes [41].

Differentially expressed transcripts were validated by analysing their expression by real-time quantitative PCR (RT-qPCR). The expression of three transcripts (MAL7P1.93 (mitochondrial ribosomal protein S8, putative), MAL7P1.175 (hypothetical protein) and PFL0055 c (protein with DNAJ domain (resa-like), putative)) was evaluated by qPCR, utilising PFL0625 c (eukaryotic translational initiation factor 3) as an endogenous control, as described previously [26].

There was a good concordance between expression levels from array analysis and those determined by qPCR (Table 3). At 2 hpt, the expression values (log_2_) for MAL7P1.93 were 1.09 and 1.29 for array and qPCR analyses respectively, and those for MAL7P1.175 were 1.89 and 2.02. Furthermore, the array expression values for the endogenous control were unchanged (log_2 _values at 2 and 6 hpt were 0.0037 and -0.0946, respectively).

**Table 3:** Comparison of expression values (log_2_)* as determined by transcriptome analysis and RT-qPCR.

PlasmoDB ID	array	RT-qPCR	array	RT-qPCR
	**2 hpt**	**2 hpt**	**6 hpt**	**6 hpt**

MAL7P1.93	1.09	1.29	1.35	2.12

MAL7P1.175 (oligo 1)	1.89	2.02	1.27	2.11
			
MAL7P1.175 (oligo 2)	1.98		1.7	

PFL0055c	1.5	3.38	2.17	2.61

eTFIII (PFL0625c)	0.0037	housekeeping	-0.0946	housekeeping

*Data numerical values were transformed to the log base 2 in order to treat gene induction and repression on an equal level mathematically.

MADIBA analyses for metabolic pathways enriched following drug perturbation indicated that several metabolic pathways were involved in the parasite's response to perturbation with dehydrobrachylaenolide. At T_2_, transcripts involved in 20 metabolic pathways were affected; at T_6_, this increased to 26. The only metabolic pathway with an appreciable p-value (in Fisher's contingency test in MADIBA) was "Biosynthesis of type II polyketide backbone", affected at T_6 _(p = 0.190). However, there is scant information regarding this pathway in the parasite. Higher p-values are expected from these analyses, since all affected transcripts at a given time point are submitted for analyses, without clustering, as described [26].

Gene Ontology (GO) analysis for genes affected by dehydrobrachylaenolide treatment, for both Biological Process and Molecular Function, were performed using MADIBA software [30]. Biological Process GO terms occurring at multiple time points (T_2 _and T_6_; at corrected p < 0.05) included chromatin silencing and dephosphorylation. Additional Biological Process GO terms included electron transport and DNA replication (T_2_) and tRNA modification (T_6_). The Molecular Function GO term chromatin binding appeared at both T_2 _and T_6_; as did histone binding. In addition, pepsin A activity (T_2_), actin binding and sugar metabolism (T_6_) were affected. The GO terms are listed in Table 4.

**Table 4:** Gene Ontology terms enriched following addition of dehydrobrachylaenolide at 2 and 6 hours post-treatment (at p < 0.05).

Time post treatment	Biological process	Molecular function	p-value	Corrected p-value (false discovery rate, p < 0.05)
	chromatin silencing		0.00056108	0.0026184
			
	dephosphorylation		0.0016571	0.0069599
			
	electron transport		0.018276	0.036552
			
	DNA replication		0.027838	0.048717
		
2 hpt		chromatin binding	0.00076337	0.0049195
		
		histone binding	0.0018045	0.0095146
		
		pepsin A activity	0.019635	0.049515

	chromatin silencing		0.0022313	0.0096688
			
	dephosphorylation		0.0064848	0.018577
			
	tRNA modification		0.012566	0.029149
	
6 hpt		chromatin binding	0.00047321	0.0031305
		
		actin binding	0.003905	0.011994
		
		sugar metabolism	0.0082591	0.022196
		
		histone binding	0.0082591	0.022196

## Discussion

The EtOAc extract of *D. anomala *subsp. *gerrardii *showed sufficient activity against *P. falciparum *to warrant further investigation. Compound 1, dehydrobrachylaenolide, isolated as a major component of this extract, was subsequently screened for its anti-plasmodial activity and found to exhibit some activity against a chloroquine-sensitive D10 strain and a chloroquine-resistant K1 strain of *P. falciparum*. This is the first report of anti-malarial activity associated with this compound.

Dehydrobrachylaenolide was originally isolated from the roots of *Brachylaena transvaalensis*, from which its name was derived [20]. Several biological activities were reported for this natural product. These included inhibitory activity toward induction of intracellular adhesion molecule-1 (ICAM-1) [21] and anti-bacterial properties [42]. It was also shown to have significant anti-cancer activity in the NCI's 60-cell line panel, with a selectivity trend towards the leukaemia sub-panel [11].

The activity of dehydrobrachylaenolide against the chloroquine-sensitive strain (IC_50 _= 1865 nM) is within an order of magnitude of that of quinine (IC_50 _= 194 nM) [43]. Furthermore, against the chloroquine-sensitive strain, the compound has a therapeutic index of 9.2, which is close to the acceptable value of 10 for potential development [38].

To establish the chemical moieties responsible for the inhibition of *P. falciparum *inhibition by dehydrobrachylaenolide, synthetic analogues of dehydrobrachylaenolide were tested against *P. falciparum *utilising the pLDH assay. From these studies, it was concluded that the anti-malarial activity was mainly attributable to the conjugate acceptor properties of the methylene lactone and the methylene ketone (in the A-ring, Figure 1). The natural product possessed two readily accessible acceptor groups of this type and the absence or inaccessibility of the methylene ketone was responsible for a ten-fold enhancement in activity without concomitant increase in toxicity (or, possibly, a two-fold reduction in toxicity), suggesting that toxic liabilities were largely associated with the methylene lactone. Further examinations relating to the role of A-ring geometry and functionalization could be warranted.

Previously, the authors successfully applied a functional genomics approach to study the effects on the *P. falciparum *transcriptome of treatment with cytostatic drugs (i.e. DFMO/MDL73811 co-inhibition [44] and cyclohexylamine [26]). In the study presented here, gene expression profiling was performed on a parasitic cytocidal compound, dehydrobrachylaenolide, subsequent to its isolation, identification, demonstrated anti-malarial activity and structure-activity relationship (SAR) studies. Gene expression profiling of another sesquiterpene lactone, artesunate [41], reported 398 genes that were differentially expressed compared to untreated *P. falciparum *parasites. Further to the SAR analyses, comparison of the artesunate dataset with the dehydrobrachylaenolide dataset of 340 genes (unique genes differentially expressed at T_2 _and T_6_), resulted in only 7.65% (26 genes of the 340) overlap. This marked difference underscores their probable different modes of action, particularly since, despite being a sesquiterpene lactone, deydrobrachylaenolide does not contain the endoperoxide bridge that is characteristic of the artemisinins and key to their biological activity [45]. In contrast, the α-methylene-γ-lactone moiety, as well as the conformation of the A-ring (Figure 1) of dehydrobrachylaenolide, which is absent in the artemisinins, is significant for various biological activities of the eudesmane sesquiterpene lactones [11,46]. The structure-activity relationship studies performed for dehydrobrachylaenolide confirmed this.

## Conclusions

In this investigation the active constituent of *Dicoma anomala *subsp. *gerrardii *was isolated and identified as dehydrobrachylaenolide, an eudesmanolide-type sesquiterpene lactone. The compound demonstrated *in vitro *anti-malarial activity against a chloroquine-sensitive strain of *P. falciparum*. Structure-activity relationship studies indicated that exocyclic methylene residues on both the lactone and A-ring appear to be responsible for the observed anti-malarial activity [11]. This methylene lactone moiety is absent in the artemisinin anti-malarials, indicating a differentiated mode of action to the artemisinins. Gene expression profiling via microarray strengthened the notion that dehydrobrachylaenolide displays a different mode of action compared to artesunate [41].

In light of the development of resistance against standard anti-malarials (now also including the artemisinins [1,2]) and the quest for new drugs with novel modes of action, this finding is significant and underscores the potential of dehydrobrachylaenolide as a potential drug lead. Some recent examples of so-called pro-drugs of sesquiterpene lactones, namely ambrosin, arglabin, parthenin and parthenolide, demonstrate the value of sesquiterpene lactones as lead compounds for the development of pharmaceuticals [47-52].

It can be concluded that dehydrobrachylaenolide could play a valuable role as a drug lead. Further mechanistic studies and an *in vivo *dose response toxicity study would be necessary to gain in-depth knowledge of the structural requirements for selectivity, with respect to the desired biological activity, and systemic toxicity.

## Competing interests

The authors declare that they have no competing interests.

## Authors' contributions

JVWB performed all transcriptional analyses, its validation and analysis and contributed to drafting the manuscript, MMvdM was responsible for the background studies, isolation, structural elucidation and initial *in vitro *data interpretation of anti-malarial activity, ACvB assisted with data interpretation, drafting and finalizing the manuscript, PP drafted the natural product and *in vitro *bioassay sections of the manuscript, BGC was responsible for parasite drug treatments for morphological and gene expression experiments, EMM performed the chemical synthesis of the dehydrobrachylaenolide analogues, CP provided technical input into the manuscript and designed the synthetic study, FvH supervised the isolation and structural elucidation aspects, NRC was responsible for plant selection, and botanical/ethnomedicinal information included in this manuscript, PJS was responsible for the *in vitro *biological assay data, DTM contributed to study conceptualization, morphology profiling, and drafting the manuscript, VJM was involved in the plant selection and collection, structural elucidation and *in vitro *data interpretation. All authors read and approved the final manuscript.

## Supplementary Material

Additional file 1 **Generation of synthetic analogues**. The file contains a description of the synthesis of dehydrobrachylaenolide analogues for structure-activity relationship studies. Click here for file

Additional file 2 **Chemical and physical data (including the NMR assignments) of compound 1**. The file contains the chemical and physical data (including the NMR assignments) of compound 1 used to identify it as dehydrobrachylaenolide. Click here for file

Additional file 3 **Dose response and survival curves for dehydrobrachylaenolide assayed against *P. falciparum *and the CHO cell-line**. The file contains examples of dose-response curves of dehydrobrachylaenolide obtained for the chloroquine-sensitive strain D10 and chloroquine-resistant strain K1. Click here for file

Additional file 4 **Microarray correlation values between samples at 2, 6 and 12 hours post-treatment**. Correlation values between collated microarray samples at 2, 6 and 12 hours post-treatment. Click here for file

Additional file 5 **Differentially expressed genes identified in *P. falciparum *following dehydrobrachylaenolide treatment**. File contains lists of differentially up- and downregulated gene identifications (IDs), annotations and relative expression values at each time point following dehydrobrachylaenolide treatment. Click here for file
