# Supplementary material for: In vitro anti-plasmodial activity of Dicoma anomala subsp. gerrardii (Asteraceae): identification of its main active constituent, structure-activity relationship studies and gene expression profiling
Source: Malar J. 2011 Oct 11;10:295. doi: 10.1186/1475-2875-10-295 (PMC3200184; doi:10.1186/1475-2875-10-295)
Supplement: Additional file 1 — Generation of synthetic analogues. The file contains a description of the synthesis of dehydrobrachylaenolide analogues for structure-activity relationship studies. [file 1475-2875-10-295-S1.DOC]

**Generation of Synthetic Analogues**

(3*S*,3a*R*,5a*S*,9b*S*)-3,5a,9-trimethyl-3-(phenylselenyl)-3a,4,5,5a-tetrahydronaphtho[1,2-*b*]furan-2,8(3*H*,9b*H*)-dione

A solution of LDA [generated from n-butyllithium (1.6M in hexanes, 26.7cm^3^, 42.71mmol) and diisopropylamine (6.2cm^3^, 44.158mmol) in dry THF (73.7cm^3^)] was cooled to –78ºC. A solution of α-santonin (**3**) (3.598g, 14.727mmol) in dry THF (73.7cm^3^) was added dropwise to the LDA solution over 30 minutes, affording a deep red mixture. After stirring for 1h, phenylselenyl chloride (8.185g, 42.737mmol) in dry THF (122.7cm^3^) was added dropwise over 1h, giving a pale orange solution. This was stirred a further 1h at –78ºC, then warmed to 0ºC and stirred for 2h. Saturated aqueous ammonium chloride (150cm^3^) was then added, and the mixture stirred for 30 minutes, then partitioned and washed with ethyl acetate. Drying (MgSO_4_) and concentration to an orange oil, followed by column chromatography (30 – 50% ethyl acetate:hexane as eluent) afforded a yellow malodorous solid. Recrystallisation (dichloromethane/hexane at 0ºC) yielded a white powder (2.062g, 35%); *R*_f_ 0.39 (30% ethyl acetate: hexane); mp. 170 - 174ºC (ethyl acetate/hexane); ^1^H NMR (400 MHz, CDCl_3_) δ 7.73 – 7.57 (m, 2H), 7.52 – 7.42 (m, 1H), 7.39 – 7.22 (m, 2H), 6.73 (d, *J* 9.9, 1H), 6.24 (d, *J* 9.9, 1H), 5.23 (dd, *J* 1.3, 10.9, 1H), 2.72 (br s, 2H), 2.12 (d, *J* 1.4, 3H), 2.08 – 1.86 (m, 5H), 1.60 (s, 3H), 1.53 (tdd, *J* 3.6, 7.8, 10.1, 1H), 1.34 (s, 3H); ^13^C NMR (101 MHz, CDCl_3_) δ 185.80, 174.43, 154.67, 150.89, 137.88, 137.83, 137.80, 137.77, 129.63, 128.87, 128.83, 128.42, 125.42, 123.51, 78.98, 57.01, 48.47, 41.02, 36.98, 24.65, 21.88, 20.12, 10.63; HRMS (ESI) calculated C_21_H_22_O_3_NaSe 425.0632, found 425.0618 (M^+^ + Na^+^).

(3a*S*,5a*S*,9b*S*)-5a,9-dimethyl-3-methylene-3a,4,5,5a-tetrahydronaphtho[1,2-*b*]furan-2,8(3*H*,9b*H*)-dione (**2**)

The selenide (2.012g, 5.013mmol) in THF (50cm^3^) at 0ºC was treated with 50% hydrogen peroxide (14.7M, 0.85cm^3^, 12.533mmol) and the mixture stirred vigorously for 1h. Brine (100cm^3^) was added, and the organic components were extracted with ethyl acetate. Drying (MgSO_4_) and concentration to an orange oil, followed by column chromatography (30 – 50% ethyl acetate:hexane as eluent) afforded a bright yellow solid (0.694g, 57%); *R*_f_ 0.50 (50% ethyl acetate: hexane); ^1^H NMR (400 MHz, CDCl_3_) δ 6.73 (d, *J* 9.9, 1H), 6.27 (d, *J* 9.9, 1H), 6.25 (d, *J* 3.3, 1H), 5.58 (d, *J* 3.1, 1H), 4.79 (dq, *J* 1.2, 11.6, 1H), 2.72 (tq, *J* 3.3, 11.7, 1H), 2.27 – 2.07 (m, 1H), 2.16 (d, *J* 1.4, 3H), 1.95 (ddd, *J* 2.2, 3.8, 13.4, 2H), 1.80 (tdd, *J* 3.9, 11.9, 13.1, 1H), 1.60 (td, *J* 4.6, 13.2, 1H), 1.33 (s, 3H); ^13^C NMR (101 MHz, CDCl_3_) δ 186.16, 169.07, 154.77, 150.74, 137.36, 128.76, 125.81, 119.67, 81.34, 50.19, 41.27, 37.52, 25.07, 21.53, 10.74; HRMS (ESI) calculated C_15_H_17_O_3_Na 245.1178, found 245.1161 (M^+^ + H^+^), and C_15_H_16_O_3_Na 267.0997, found 267.0994 (M^+^ + Na^+^). The data was in accordance with literature values.^57^

(3a*S*,5a*S*,9*R*,9a*S*,9b*S*)-5a,9-dimethyl-3-methylene-3a,4,5,5a,9,9a-hexahydronaphtho[1,2-*b*]furan-2,8(3*H*,9b*H*)-dione (**6**)

The diselenide derived from base mediated addition of phenylselenyl chloride to compound (**4**) (0.294g, 0.524mmol) in THF (6cm^3^) at 0ºC was treated with 50% hydrogen peroxide (14.7M, 0.089cm^3^, 1.31mmol) and the mixture stirred vigorously for 1h. Similar workup, followed by column chromatography (30% ethyl acetate:hexane as eluent) afforded a white solid (65.1mg, 50%); *R*_f_ 0.50 (30% ethyl acetate:hexane); ^1^H NMR (400 MHz, CDCl_3_) δ 6.72 (1 H, d, *J* 9.9), 6.12 (1 H, d, *J* 3.2), 5.92 (1 H, d, *J* 9.9), 5.45 (1 H, d, *J* 3.1), 3.99 (1 H, t, *J* 10.9), 2.60 (1 H, dq, *J* 6.8, 12.5), 2.65 – 2.52 (1 H, m), 2.17 – 2.04 (2 H, m), 1.83 – 1.64 (2 H, m), 1.62 (1 H, br m), 1.41 (3 H, d, *J* 6.9), 1.22 – 1.11 (3 H, m); ^13^C NMR (101 MHz, CDCl_3_) δ 200.38, 170.08, 157.95, 138.13, 126.52, 117.41, 81.88, 52.04, 50.02, 41.88, 38.39, 36.98, 21.03, 19.13, 14.48; HRMS (ESI) calculated C_15_H_18_NaO_3_ 269.1154, found 269.1107 (M^+^ + Na^+^) and calculated C_15_H_19_O_3_ 247.1334, found 247.1297 (MH^+^).

(9b*S*)-5a,9-Dimethyl-3-methyleneoctahydro-1*H*-cyclopenta[a]naphthalene-2,8(3*H*,9b*H*)-dione (**5**)

To a solution of freshly prepared lithium diisopropylamide (7.49 mmol) in tetrahydrofuran (40 mL), stirred at -77 ºC, under a nitrogen gas atmosphere, was added, by syringe, a solution of (9b*S*)-3,5a,9-trimethyloctahydronaphtho[1,2-*b*]furan-2,8(3*H*,9b*H*)-dione (1.50 g, 5.99 mmol) in freshly distilled anhydrous tetrahydrofuran (20 mL). The resulting solution was allowed to stir at the said temperature for 1 hour, after which a solution of phenylselenyl chloride (1.44 g, 7.49 mmol) in freshly distilled anhydrous tetrahydrofuran (20 mL) was slowly added, by syringe. The resultant dark orange solution was allowed to warm up to room temperature over 2 hours. The reaction mixture was then poured into a separating funnel containing a saturated NH_4_Cl_(aq)_ (200 mL) and ethyl acetate (200 mL), the layers separated, and the organic layer was dried over MgSO_4_, filtered through celite and the filtrate concentrated on a rotary evaporator, under reduced pressure. The crude product dark orange brown oil thus isolated was purified by silica gel column chromatography, using ethyl acetate in hexanes (30% v/v) as eluent to give [(9b*R*)-3,5a,9-trimethyl-3-(phenylselanyl)octahydronaphtho[1,2-*b*]furan-2,8(3*H*,9b*H*)-dione] as a colorless solid (1.58g, 65%). This was not characterized but immediately subjected to oxidative elimination as follows:

[(9bR)-3,5a,9-trimethyl-3-(phenylselanyl)octahydronaphtho[1,2-*b*]furan-2,8(3H,9bH)-dione] (1.58 g,3.89 mmol) was dissolved in an ethyl acetate-methanol mixture (1:1, 100 mL) and to this solution, stirred at 0 ºC, was added H_2_O_2(aq)_, (35%; 40 mL) and the reaction mixture allowed to stir at room temperature for 4 hours. Thin layer chromatographic analysis showed total consumption of starting material and thus the reaction mixture was poured into a separating funnel containing ethyl acetate (200 mL) and washed with a saturated NaHCO_3(aq)_ solution (3 × 150 mL). The organic layer was dried over MgSO_4_, filtered through celite and the filtrate concentrated on a rotary evaporator, under reduced pressure. The white waxy solid thus isolated was recrystallized from Ethyl acetate-Hexanes to give the title compound, (9b*S*)-5a,9-Dimethyl-3-methyleneoctahydro-1*H*-cyclopenta[a]naphthalene-2,8(3*H*,9b*H*)-dione, as white granules.

^1^H NMR (400 MHz, CDCl_3_) δ 6.09 (s, 1H), 5.43 (s, 1H), 3.90 (td, *J* = 3.4, 11.1, 1H), 2.65 – 2.37 (m, 4H), 2.17 – 2.00 (m, 1H), 1.87 – 1.48 (m, 3H), 1.51 – 1.07 (m, 8H).

^13^C NMR (100 MHz, CDCl_3_) δ 211.0, 170.2, 138.5, 117.3, 83.2, 54.0, 49.8, 44.7, 40.6, 39.8, 37.2, 36.6, 21.4, 18.3, 13.8.

(3*S*,3a*S*,5a*S*,9*R*,9a*S*,9b*S*)-3,5a,9-trimethyl-octahydronaphtho[1,2-*b*]furan-2,8(3*H*,9b*H*)-dione (**4**)

Santonin (**3**) (5.026g, 20.406mmol), %Pd-C (4.536g, ~1 mass eq.) and ethanol (50cm^3^) were mixed under hydrogen atmosphere (1 atm) for 48h. The catalyst was filtered off over celite, washed with ethanol and acetone, and concentrated to a white solid. Column chromatography (12% ethyl acetate: hexane – ethyl acetate gradient) afforded a pure white solid, a mixture of the two *cis* isomers. Recrystallisation afforded a white solid (1.342g, 26%); *R*_f_ 0.66 (50% ethyl acetate: hexane); ^1^H NMR (400 MHz, CDCl_3_) δ 3.92 (1 H, t, *J* 10.2), 2.60 – 2.48 (2 H, m), 2.44 (1 H, ddd, *J* 2.5, 5.6, 15.6), 2.28 (1 H, dq, *J* 6.9, 13.8), 1.91 – 1.84 (1 H, m), 1.77 (1 H, ddd, *J* 2.5, 6.9, 13.3), 1.70 (1 H, dd, *J* 4.6, 8.1), 1.68 – 1.63 (1 H, m), 1.63 – 1.54 (3 H, m), 1.39 – 1.28 (1 H, m), 1.25 (3 H, d, *J* 6.6), 1.22 (3 H, d, *J* 6.9), 1.19 (3 H, s); ^13^C NMR (101 MHz, CDCl_3_) δ 211.50, 179.08, 83.07, 53.43, 52.81, 44.92, 40.66, 40.59, 40.11, 37.32, 36.57, 23.03, 18.36, 13.81, 12.44; HRMS (ESI) calculated C_15_H_22_NaO_3_ 273.1467, found 273.1479 (M^+^ + Na^+^).
