# Supplementary material for: In vitro anti-plasmodial activity of Dicoma anomala subsp. gerrardii (Asteraceae): identification of its main active constituent, structure-activity relationship studies and gene expression profiling
Source: Malar J. 2011 Oct 11;10:295. doi: 10.1186/1475-2875-10-295 (PMC3200184; doi:10.1186/1475-2875-10-295)
Supplement: Additional file 2 — Chemical and physical data (including the NMR assignments) of compound 1. The file contains the chemical and physical data (including the NMR assignments) of compound 1 used to identify it as dehydrobrachylaenolide. [file 1475-2875-10-295-S2.DOC]

**Chemical and physical data (including the NMR assignments) of compound 1**

| Common name: | Dehydrobrachylaenolide |
| --- | --- |
| IUPAC name: | (3a*S*,5a*S*,9a*R*,9b*S*)-5a-methyl-3,9-dimethylidene-4,5,9a,9b-tetrahydro-3aH-naphtho[7,8-d]furan-2,8-dione |
| Molecular formula: | C_15_H_16_O_3_ |
| ESI-MS, m/z: | 245.2 [M+H]^+^ |
| Melting point: | 223-224°C (mp 225°C, [16]) |
| Optical rotation: | [α]^24^*_D_* +68°(*c*= 0.50, CHCl_3_); ([α]^24^*_D_* +67.9°, *c*= 0.16, CHCl_3_ [17]) |
| UV, λ_max_ (nm): | 209; 244 |
| ^1^H NMR (400 MHz, CDCl_3_), *δ*_H_: | 1.02 (3H, s, H-14); 1.67 (2H, m, H-7, Ha-8); 1.81 (1H, m, H-9), 2.1 (1H, m, H-8); 2.6 (1H, m, H-7); 2.98 (1H, ddd, J= 2.2; 2.3; 10.9 Hz, H-5); 4.07 (1H, dd, J= 10.7; 10.8 Hz, H-6); 5.43 (1H, d, J= 3.1 Hz, H-13); 5.68 (1H, ddd, J= 0.9; 1.0; 2.4 Hz, H-15), 5.99 (1H, d, J= 9.9 Hz, H-2); 6.1 (1H, d, J= 3.2 Hz, H-13); 6.24 (1H, dd, J= 1.0; 2.2 Hz, H-15); 6.76 (1H, d, J= 10.0 Hz, H-1). |
