# Supplementary material for: In vitro anti-plasmodial activity of Dicoma anomala subsp. gerrardii (Asteraceae): identification of its main active constituent, structure-activity relationship studies and gene expression profiling
Source: Malar J. 2011 Oct 11;10:295. doi: 10.1186/1475-2875-10-295 (PMC3200184; doi:10.1186/1475-2875-10-295)
Supplement: Additional file 3 — Dose response and survival curves for dehydrobrachylaenolide assayed against P. falciparum and the CHO cell-line. The file contains examples of dose-response curves of dehydrobrachylaenolide obtained for the chloroquine-sensitive strain D10 and chloroquine-resistant strain K1. [file 1475-2875-10-295-S3.DOC]

**Dose response and survival curve examples**
