# Supplementary material for: In vitro anti-plasmodial activity of Dicoma anomala subsp. gerrardii (Asteraceae): identification of its main active constituent, structure-activity relationship studies and gene expression profiling
Source: Malar J. 2011 Oct 11;10:295. doi: 10.1186/1475-2875-10-295 (PMC3200184; doi:10.1186/1475-2875-10-295)
Supplement: Additional file 4 — Microarray correlation values between samples at 2, 6 and 12 hours post-treatment. Correlation values between collated microarray samples at 2, 6 and 12 hours post-treatment. [file 1475-2875-10-295-S4.DOC]

**Microarray correlation values between samples at 2, 6 and 12 hours post-treatment**

| **Slides** | **Correlation values** |
| --- | --- |
| **Control 2h** |  |
| Technical replicates 1 | 0.93 |
| Technical replicates 2 | 0.61 |
| Biological replicate 1 | 0.64 |
| Biological replicate 2 | 0.59 |
| Biological replicate 3 | 0.61 |
| Biological replicate 4 | 0.58 |
| **Control 6h** |  |
| Technical replicates | 0.97 |
| Biological replicate 1 | 0.93 |
| Biological replicate 2 | 0.92 |
| **Control 12h** |  |
| Technical replicates | 0.97 |
| Biological replicate 1 | 0.96 |
| Biological replicate 2 | 0.96 |
| **Ref31 2h** |  |
| Technical replicates | 0.90 |
| Biological replicate 1 | 0.60 |
| Biological replicate 2 | 0.61 |
| **Ref31 6h** |  |
| Technical replicates | 0.97 |
| Biological replicate 1 | 0.88 |
| Biological replicate 2 | 0.88 |
| **Ref31 12h** |  |
| Technical replicates | 0.87 |
| Biological replicate 1 | 0.84 |
| Biological replicate 2 | 0.93 |
